# Literary Notes

**Published:** 1896-06

**Authors:** 


### Literary Notes.

THE *National Medical Review*, of Washington, is now "the official journal of the Medical Society of the District of Columbia." All papers and discussions given before that society will be published in the official journal. This society now numbers about 300 active members, and includes a number of the most prominent medical men in the army and navy. The editor announces that his journal will be enlarged and otherwise greatly improved in order to carry into effect this new arrangement.

---

WEIR'S *Index to the Medical Press*, Vol. I., No. 1, is at hand. It is a monthly journal devoted to medical bibliography, at present including in its scope only American publications. It is attractively printed, contains a list of the current journals referred to and an index of medical literature not only by title but by author as well. Its moderate price places it within the reach of all. Frank Weir & Co., publishers, New York. $3.00 per annum.

---

THE NEWER REMEDIES, by Virgil Coblentz, A. M., Phil. D., F. C. S., etc., is a reference manual of the newer remedies, together with their sources, methods of preparation, tests, incompatibles, medicinal properties and doses. It is a valuable little work which any physician may obtain free by writing to McKesson & Robbins, wholesale druggists, 91 Fulton street, New York.

---

*The Woman's Hospital Surgical and Gynecological Clinic* will be issued from St. Louis on June 1st, (*American Therapist*,) Dr. George F. Hulbert, president of the St. Louis Woman's Hospital, editor and publisher. Subscribers will be sought primarily among country physicians who combine surgery with general practice, but city doctors may also subscribe.

---

LEA BROTHERS & Co., publishers, Philadelphia and New York, announce the early issue of A Treatise on Surgery by American authors, edited by Roswell Park, M. D. The work is to be issued in two volumes, royal octavo. Among the contributors are John Parmenter, M. D., of Buffalo, and Chauncey P. Smith, M. D., of Buffalo.

---

THE New York Pharmacal Association calls attention to the merits of Lactopeptine in one of the prettiest booklets seen for many a day, entitled The Test of Time, which is printed in old English text, illuminated, on imitation cloth, with cloth binding.

---

THE Drevet Manufacturing Company, of New York, has secured an injunction against Dr. A. P. Beach, of Seville, O., restraining him from using their trade-mark, Glycozone.

P. Blakiston, Son & Co., 1012 Walnut street, Philadelphia, have lately issued a portrait catalogue which is a beautiful guide to their publications on medicine, dentistry, pharmacy, chemistry and allied subjects.

---

The Rio Chemical Company have recently issued a handsome pamphlet on Urethral Diseases, containing clinical reports of the use of S. H. Kennedy's extract of pinus canadensis.

